# The International Bathymetric Chart of the Arctic Ocean Version 5.0

**DOI:** 10.1038/s41597-024-04278-w

**Published:** 2024-12-21

**Authors:** Martin Jakobsson, Rezwan Mohammad, Marcus Karlsson, Silvia Salas-Romero, Florian Vacek, Florian Heinze, Caroline Bringensparr, Carlos F. Castro, Paul Johnson, Juliet Kinney, Sara Cardigos, Michael Bogonko, Daniela Accettella, David Amblas, Lu An, Aileen Bohan, Angelika Brandt, Stefan Bünz, Miquel Canals, José Luis Casamor, Bernard Coakley, Natalie Cornish, Seth Danielson, Maurizio Demarte, Davide Di Franco, Mary-Lynn Dickson, Boris Dorschel, Julian A. Dowdeswell, Simon Dreutter, Alice C. Fremand, John K. Hall, Bryan Hally, David Holland, Jon Kuk Hong, Roberta Ivaldi, Paul C. Knutz, Diana W. Krawczyk, Yngve Kristofferson, Galderic Lastras, Caroline Leck, Renata G. Lucchi, Giuseppe Masetti, Mathieu Morlighem, Julia Muchowski, Tove Nielsen, Riko Noormets, Andreia Plaza-Faverola, Megan M. Prescott, Autun Purser, Tine L. Rasmussen, Michele Rebesco, Eric Rignot, Søren Rysgaard, Anna Silyakova, Pauline Snoeijs-Leijonmalm, Aqqaluk Sørensen, Fiammetta Straneo, David A. Sutherland, Alex J. Tate, Paola Travaglini, Nicole Trenholm, Esmee van Wijk, Luke Wallace, Josh K. Willis, Michael Wood, Mark Zimmermann, Karl B. Zinglersen, Larry Mayer

**Affiliations:** 1 Department of Geological Sciences, Stockholm University, Stockholm, Sweden; 2 Utrecht University, Utrecht, Netherlands; 3 Center for Coastal and Ocean Mapping, University of New Hampshire, Durham, NH USA; 4 OGS National Institute of Oceanography and Applied Geophysics, Sgonico, Italy; 5 CRG Marine Geosciences, Department of Earth and Ocean Dynamics, University of Barcelona, Barcelona, Spain; 6 College of Surveying and Geo-informatics, Tongji University, Shanghai, China; 7 INFOMAR, Geological Survey Ireland, Dublin, Ireland; 8 Senckenberg Research Institute and Natural History Museum & Goethe University, Frankfurt am Main, Germany; 9 UiT – The Arctic University of Norway, Tromsø, Norway; 10 Reial Acadèmia de Ciències i Arts de Barcelona (RACAB), Barcelona, Spain; 11 Institut d’Estudis Catalans (IEC), Secció de Ciències i Tecnologia, Barcelona, Spain; 12 Geophysical Institute, University of Alaska, Fairbanks, AK USA; 13 Alfred Wegener Institute, Helmholtz Centre for Polar and Marine Research, Bremerhaven, Germany; 14 College of Fisheries and Ocean Sciences, University of Alaska, Fairbanks, AK USA; 15 Italian Hydrographic Institute, Genoa, Italy; 16 Geological Survey of Canada, Dartmouth, Nova Scotia Canada; 17 Scott Polar Research Institute, University of Cambridge, Cambridge, UK; 18 UK Polar Data Centre, British Antarctic Survey, Cambridge, UK; 19 Geological Survey of Israel, Jerusalem, Israel; 20 CSIRO Environment, Aspendale, Victoria, Australia; 21 School of Geography, Planning and Spatial Science, University of Tasmania, Sandy Bay, Australia; 22 Courant Institute of Mathematical Sciences, New York University, New York, NY USA; 23 Korea Polar Research Institute, Incheon, Korea; 24 Geological Survey of Denmark and Greenland, Copenhagen, Denmark; 25 Greenland Institute of Natural Resources, Nuuk, Greenland; 26 Department of Earth Science, University of Bergen, Bergen, Norway; 27 Department of Meteorology, Stockholm University, Stockholm, Sweden; 28 Danish Geodata Agency, Danish Hydrographic Office, Ålborg, Denmark; 29 Department of Earth Sciences, Dartmouth College, Hanover, NH USA; 30 University Centre in Svalbard, Longyearbyen, Svalbard Norway; 31 Lynker Technologies, Seattle, WA USA; 32 NOAA National Marine Fisheries Service, Alaska Fisheries Science Center, Seattle, USA; 33 Department of Earth System Science, University of California, Irvine, CA USA; 34 Radar Science and Engineering Section, Jet Propulsion Laboratory, California Institute of Technology, Pasadena, CA USA; 35 Department of Civil and Environmental Engineering, University of California, Irvine, CA USA; 36 Université Grenoble Alpes, CNRS, IRD, INP, 38400 Grenoble, Isère France; 37 HUB Ocean, Oslo, Norway; 38 Department of Ecology, Environment and Plant Sciences, Stockholm University, Stockholm, Sweden; 39 Scripps Institution of Oceanography, University of California San Diego, La Jolla, CA USA; 40 Department of Earth Sciences, University of Oregon, Eugene, OR USA; 41 British Antarctic Survey, Cambridge, UK; 42 Canadian Hydrographic Service, Ottawa, Canada; 43 Ocean Research Project, Annapolis, MD USA; 44 CSIRO Environment, Hobart, Tasmania Australia; 45 Australian Antarctic Program Partnership, University of Tasmania, Hobart, Tasmania Australia; 46 Moss Landing Marine Labs, San Jose State University, San Jose, CA USA

**Keywords:** Ocean sciences, Geomorphology

## Abstract

Knowledge about seafloor depth, or bathymetry, is crucial for various marine activities, including scientific research, offshore industry, safety of navigation, and ocean exploration. Mapping the central Arctic Ocean is challenging due to the presence of perennial sea ice, which limits data collection to icebreakers, submarines, and drifting ice stations. The International Bathymetric Chart of the Arctic Ocean (IBCAO) was initiated in 1997 with the goal of updating the Arctic Ocean bathymetric portrayal. The project team has since released four versions, each improving resolution and accuracy. Here, we present IBCAO Version 5.0, which offers a resolution four times as high as Version 4.0, with 100 × 100 m grid cells compared to 200 × 200 m. Over 25% of the Arctic Ocean is now mapped with individual depth soundings, based on a criterion that considers water depth. Version 5.0 also represents significant advancements in data compilation and computing techniques. Despite these improvements, challenges such as sea-ice cover and political dynamics still hinder comprehensive mapping.

## Abstract (summary)

**Table:** 

Measurement(s)	Depth
Technology Type(s)	Digital curation
Factor Type(s)	Geographic location
Sample Characteristic - Environment	Ocean floor
Sample Characteristic - Location	Arctic Ocean

## Background & Summary

Bathymetry, the study of seafloor depth, is the foundation for a broad range of marine activities such as scientific research, safety of navigation, environmental monitoring, spatial planning, underwater construction and ocean exploration^1^. Bathymetric mapping has been particularly difficult in the central Arctic Ocean due to the perennial sea-ice cover, restricting the vessels capable of acquiring data to icebreakers and under-ice vehicles, including submarines^2^. In part for this reason, bathymetry and other geophysical mapping data have also been acquired occasionally from platforms or stations drifting with the pack ice^3–5^. Furthermore, while predicted bathymetry from satellite altimetry has supported bathymetric compilations in most other parts of the world’s oceans^6^, the sea-ice cover, complex seabed geology and thick sediment cover over large areas in the central Arctic Ocean have limited the applicability of this method, although notable efforts have been made^7^.

**Fig. 1:**
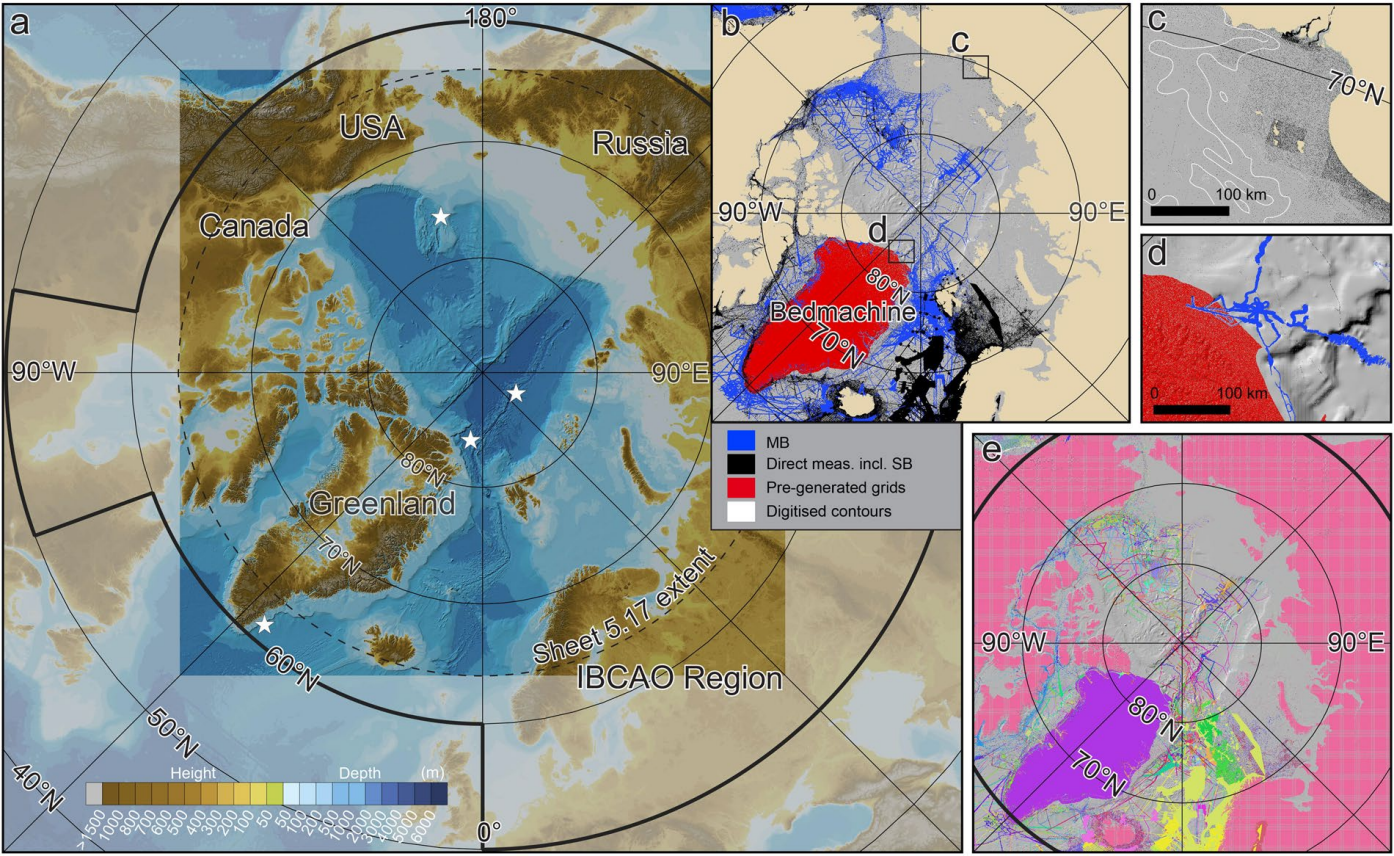
Overview maps illustrating Arctic Ocean bathymetry and source data for the compilation of IBCAO 5.0. (**a**) Bathymetry based on IBCAO 5.0. Two versions are available: one with under-ice topography of Greenland (shown), and another with the ice-sheet surface topography, both based on BedMachine Version 5 DMT^180^. The bold black line shows the Seabed 2030 Arctic region, for which a geographic DTM is produced and contributed to the global GEBCO DTM. The square region shown in brighter colours represents the more limited extent of the IBCAO DTM. White stars show the locations of detailed comparison between IBCAO 5.0 and 4.0 in Fig. 9. (**b**) Source data displayed based on the mapping method (MB = Multibeam; SB = Singlebeam). (**c**) Close-up of the East Siberian Sea depicting soundings from charts (in black) and digitised contours (in white). The nodes of the digitised contours, utilised in the gridding process, may be challenging to discern due to their sparse density. To enhance visibility, several contours are presented as polygons in white. (**d**) Close-up of North Greenland showing a part of the least mapped area of the Arctic Ocean. (**e**) Source data displayed as individual data sets using different colours. Note that the high resolution of the IBCAO 5.0 gridded products precludes displaying fine details in overview figures. For detailed information, readers are referred to the downloadable grids.

In 1997, a new project named the International Bathymetric Chart of the Arctic Ocean (IBCAO) was initiated in St. Petersburg, Russia, to accelerate Arctic Ocean mapping^8^. The project’s goal was to assemble all available Arctic bathymetric data and compile an updated map of the Arctic Ocean floor, given the growing recognition of significant errors in existing maps^9^. The region of interest was confined to the extent of the Arctic Ocean Sheet 5.17 (64°N) published in 1979 in the General Bathymetric Chart of the Oceans (GEBCO) chart series^10^ (Fig. 1). However, by 1997 it was recognized that there was much greater value in a gridded digital product rather than an updated paper chart. Therefore, IBCAO focused on producing a gridded Digital Terrain Model (DTM) of seafloor depths. The DTM was produced in the form of a Cartesian grid in a Polar Stereographic projection, with a true scale at 75°N (EPSG: 3996) in accordance with GEBCO Sheet 5.17 (Fig. 1). Following the first beta release at the American Geophysical Union Fall Meeting in San Francisco 1999^11^, four major versions (1.0–4.0) of the IBCAO grid have been published and made available for public download alongside descriptive papers^12–14^. Version 1.0 was released with a grid-cell size of 2.5 × 2.5 km, Version 2.0 at 2 × 2 km, Version 3.0 at 500 × 500 m and Version 4.0 at 200 × 200 m. Here we describe the new IBCAO Version 5.0, henceforth referred to as IBCAO 5.0, released at a grid-cell size of 100 × 100 m, and the compilation methods and source data used to produce it.

In 2017, the *Nippon Foundation-GEBCO-Seabed 2030* project was launched with the ambitious goal of mapping all of the World’s oceans by the year 2030^15,16^. To achieve this, four regional centres were established, each tasked with the responsibility of gathering and compiling the available bathymetric data from their specific ocean regions. Figure 1 shows the Arctic region as defined by Seabed 2030, with Stockholm University and the University of New Hampshire jointly hosting the Seabed 2030 Arctic Regional Centre. Given that the IBCAO region fell within the Arctic Regional Centre’s responsibility area (Fig. 1), it was a natural choice for the centre to take the leading role in the compilation of IBCAO. IBCAO is maintained as a separate scientific product because the Arctic research community, along with other users of IBCAO, have a sustained interest in using a Polar Stereographic DTM. This complements Seabed 2030’s primary global product, the GEBCO grid, which is currently released as a geographic DTM at a resolution of 15 × 15 arc seconds^17^. This is analogous to the parallel production of the International Bathymetric Chart of the Southern Ocean (IBCSO), which was recently published as Version 2.0 of a Polar Stereographic DTM (EPSG: 9354) at a grid-cell size of 500 × 500 m^18^, along with the inclusion of IBCSO DTM in the Seabed 2030 GEBCO grid.

**Fig. 2:**
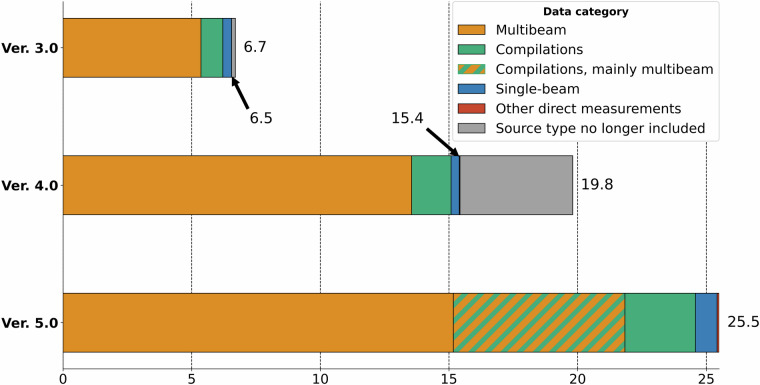
Comparison between the three main source data categories in IBCAO 3.0, 4.0 and 5.0. Note that the grey sections of the bars for IBCAO 3.0 and 4.0 represent source data types we no longer count when calculating mapping coverage. See Table 2 for definition of the data categories. A large segment of the “compilations” data category is likely composed of multibeam measurements, although only a rough estimation is currently available.

It is difficult to precisely compare the increase in coverage from IBCAO 4.0 to 5.0 as we have refined the statistical calculation method, for example, by being more stringent about which types of data are counted to map an area. A major difference is how we previously, in IBCAO 4.0, assumed the extent of the BedMachine^19^ compilation of Greenland waters near the coast as mapped. Instead, we now extract and count only the underlying source data sets. This ensures that all interpolated data points are excluded where depth soundings have not been made. Originally, IBCAO 4.0 was estimated to contain bathymetric data mapping 19.8%^14^ of the larger Seabed 2030 Arctic area shown in Fig. 1 (black line in subfigure a.). However, using the current stricter statistical method would yield an estimate of 15.4% coverage. The Seabed 2030 area in IBCAO 5.0 is calculated as 25.5% (Fig. 2), which equates to an increase in mapping coverage of about 1.4 × 10^6^ km^2^, an area slightly larger than three times the size of Sweden. The more limited IBCAO DTM region is constrained to 25.7% coverage by direct depth measurements in Version 5.0. The multibeam bathymetry coverage is 15.2% and 17.9%, for the Seabed 2030 Arctic region and the more limited IBCAO DTM area, respectively. However, a considerable amount of multibeam data is included in the data category “compilations”, which consists of a mixture of various direct measurement methods, although we are currently unable to extract the exact proportion due to lack of metadata. We estimate that the multibeam coverage exceeds 20% in both the larger Seabed 2030 Arctic area and the IBCAO DTM, with roughly 6.7% of the 9.4% coverage from compilation measurements likely resulting from multibeam bathymetry. A definitive coverage statistic cannot be derived due to issues of identifying the measurement method for the data that have been provided merged together in compilations.

The above depth data coverage estimations take the Seabed 2030 variable grid-cell size scheme by depth into account (see^20^: 0–1500 m depth: 100 × 100 m; 1500–3000 m depth: 200 × 200 m; 3,000–5,750 m depth: 400 × 400 m). This implies that, in order to be considered mapped within the 0–1500 m depth band, each 100 × 100 m grid cell must contain at least one depth measurement. Similarly, for the 1,500–3,000 m depth band, each 200 × 200 m grid cell must contain at least one depth measurement, and so forth.

The calculation of the area or number of grid cells constrained by direct depth measurement can be performed in different ways, yielding distinctly different results. For example, the calculated coverage will differ if it is calculated at a fixed resolution of 100 × 100 m irrespective of water depth compared to using the Seabed 2030 variable resolution scheme by depth. Additionally, the calculated coverage for a grid with a cell size of 100 × 100 m yields a lower percentage than the equivalent calculation for a 200 × 200 m grid as many datasets have lower resolutions than 100 × 100 m. This variability illustrates why Seabed 2030 adopted a standard method for calculating mapping coverage using a defined variable grid-cell size scheme by depth, allowing for consistent monitoring of the progression in mapping the World’s oceans.

**Fig. 3:**
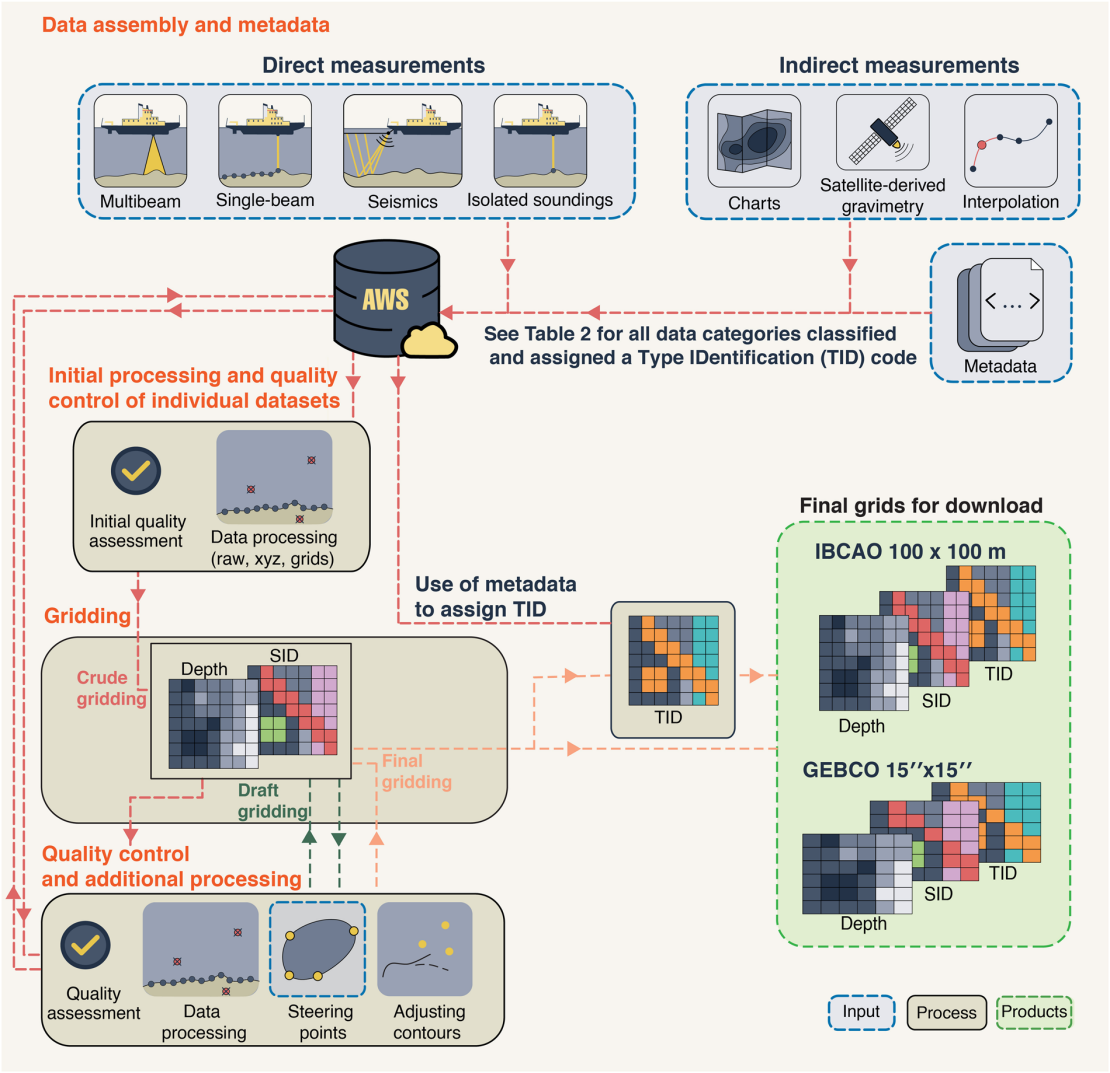
Flow chart of the major steps involved in compiling the IBCAO 5.0 grid. The orange headings correspond to sections within Methods describing the main compilation procedures. AWS: Amazon Web Services; TID: Type Identification; SID: Source Identification.

Additionally, improvements in the methods used to compile IBCAO 5.0 compared to 4.0 include more efficient use of Python routines and distributed computing in a cloud environment, along with the integration of additional metadata. This permits more detailed statistics on, for example, the type of bathymetric mapping methods, data originators, and platforms. The general flow chart is shown in Fig. 3 and the included major steps are further described under methods.

While the IBCAO DTM will continue to undergo updates and improvements as part of the Seabed 2030 project, significant challenges persist in achieving a complete map of the Arctic Ocean. A fundamental challenge is the perennial sea-ice cover, which constitutes a substantial obstacle to data collection from ships. Consequently, efficient and systematic mapping over larger sea-ice-covered areas requires the use of submarines or autonomous underwater vehicles (AUVs)^21^. A new program, similar to the Scientific Ice Expeditions (SCICEX)^2,22,23^, but with the submarines and AUVs equipped with multibeam echosounders, would be required to fully map regions with the most difficult sea-ice conditions (i.e. north of Greenland and the Canadian Arctic Archipelago). Alternatively, the development of a new generation of long-range AUVs capable of navigating under sea ice could also address these needs. AUVs are also the primary devices able to acquire bathymetry below ice-shelf cavities, which is increasingly important for understanding ice-ocean interactions and improving projections of future sea-level rise. For the same reasons, bathymetry is needed from ice-choked fjords, such as those in Greenland where Sermeq Kujalleq (Jakobshavn glacier) and Helheim glacier drain. Another challenge is the present political dynamics in the central Arctic Ocean that create obstacles to open data sharing between all nations, and sometimes restrictions on the collection of new data. All this complicates collaborative efforts for comprehensive mapping.

## Methods

The procedures involved in producing the IBCAO 5.0 DTM, the Arctic contribution to the GEBCO 15 × 15 arc second geographic grid and the source data coverage grids are illustrated in Fig. 3. The critical steps are described further below.

### Data assembly and metadata

The initial step in the compilation of the IBCAO grid consists of gathering data and critical metadata from the bathymetry providers. An interface for data upload has been developed which also facilitates providers to include the minimum metadata required for our gridding and calculation of basic statistics. These metadata details are shown in Table 1. The “Data category” entry provides information on the type of mapping methods used to gather the bathymetric data. The classification of mapping methods follows the so-called Type Identification (TID) code established within the Seabed 2030/GEBCO community (Table 2). This code has been uniformly adopted by other Seabed 2030 Regional Centres and is presented as supplementary information for the global GEBCO 15 × 15 arc second DTM^17^. Additional metadata required from providers includes the organisation which has collected or owns the data, the coordinate system of each dataset, and a “priority” rank, which is used in the gridding and statistical procedure to decide the ordering of different overlapping data sets, which is described further below. Several other metadata fields are used in the grid compilation process. As the list of datasets contains more than 6,000 records, the metadata system is needed to track each dataset and its progress throughout the compilation process. A metadata field is therefore included that describes the processing status of each dataset. This field is crucial for determining in which “variant” of the grid the dataset will be included. The grid “variants” are described further below. Several datasets are edited numerous times and each revision is tracked and documented in the metadata system, describing the updates and differences from the previous revision. This documentation is vital to minimise human error in managing all datasets and also keeping track of what remains to be done in order to finalise the processing of a specific dataset.

**Table 1:** Key entries in the provider contribution form for uploading bathymetric data.

**Dataset name**	The name of the dataset. This could be, for example, the vessel name, cruise name or number, the survey location, or other identifying information.
**Data category** (**TID**)	GEBCO type identifier code (TID), for example, multibeam, single beam. Table 2 shows the list of categories.
**Acquisition organisation**	Organisation(s) that collected or owns the data.
**Coordinate system**	The dataset coordinate system, provided by an EPSG code.
**Priority**	Determines which dataset to use in the case of overlapping data in the same grid cell through its quality or TID code.
**Contact information**	Name, Email, Organisation.
**Personal information consent/Data consent/Authority consent**	Consent that information about the provider can be stored in our database in AWS, consent that the data can be used in IBCAO, GEBCO and IBCSO, and that the provider has the authority to provide the data.
**DCDB consent**	Consent to provide the uploaded data to the IHO Data Center for Digital Bathymetry (IHO/DCDB), where the individual datasets can be downloaded. (IBCAO is not a repository for the provided bathymetric datasets, and does not distribute them further).

**Table 2:** Type Identification (TID) code for mapping methods used to gather the provided 277 bathymetric data. The TID codes were decided within the Seabed 2030/GEBCO community and implemented in the compilation of the global GEBCO DTM.

TID	Definition
0	Land
	**Direct measurements**
10	Single beam - depth value collected by a single beam echo-sounder
11	Multibeam - depth value collected by a multibeam echo-sounder
12	Seismic - depth value collected by seismic methods
13	Isolated sounding - depth value that is not part of a regular survey or track line
14	ENC sounding - depth value extracted from an Electronic Navigation Chart (ENC)
15	Lidar - depth derived from a bathymetric lidar sensor
16	Depth measured by optical light sensor
17	Combination of direct measurement methods
	**Indirect measurements**
40	Predicted based on satellite-derived gravity data - depth value is an interpolated value guided by satellite-derived gravity data
41	Interpolated based on a computer algorithm - depth value is an interpolated value based on a computer algorithm (e.g. Generic Mapping Tools)
42	Digital bathymetric contours from charts - depth value taken from a bathymetric contour data set
43	Digital bathymetric contours from ENCs - depth value taken from bathymetric contours from an Electronic Navigation Chart (ENC)
44	Bathymetric sounding - depth value at this location is constrained by bathymetric sounding(s) within a gridded data set where interpolation between sounding points is guided by satellite-derived gravity data
45	Predicted based on helicopter/flight-derived gravity data
46	Depth estimated by calculating the draft of a grounded iceberg using satellite-derived freeboard measurement
	**Unknown**
70	Pre-generated grid - depth value is taken from a pre-generated grid that is based on mixed source data types, e.g. single beam, multibeam, interpolation etc.
71	Unknown source - depth value from an unknown source
72	Steering points - depth value used to constrain the grid in areas of poor data coverage

### Initial data processing and quality control of individual datasets

All datasets submitted or collected for use in IBCAO undergo multiple rounds of quality control checks and processing before being incorporated into the final grid products. All revisions of a dataset are tracked and stored so it is possible to revert to a previous version if necessary. While we encourage contributions of DTMs and XYZ point clouds from individual surveys, we sometimes receive raw multibeam data in their native format. These raw datasets are processed using Qimera (Version 2.6.2), a hydrographic data processing software produced by QPS. All other processed datasets received, for example, DTMs in raster formats and XYZ files of direct measurements, are initially reviewed using Qimera or Caris Base Editor (Version 5.1) to ensure that the geospatial metadata, such as map projection and resolution, are correctly specified for each dataset. In addition, some outliers may still be present in the contributed processed datasets, and the overall data quality can vary depending on the original purpose of data acquisition. For instance, transit data may contain artefacts due to inadequate sound velocity control, resulting in noticeable refractions of the outer beams^24^.

Part of our initial processing is splitting the datasets into several smaller subsets, each with different spatial resolution, when the difference in minimum and maximum depth coverage is too large to account for the diameter of an echosounder’s footprint *D*_*f*_ in metres on the seafloor that will roughly increase by

1 $${D}_{f}=2H\times tan(\frac{\alpha }{2}),$$

where *H* is the water depth in metres and α is the beam width in degrees of the echosounder. Given this, the resolution decreases with depth implying that we do not need to maintain the same grid-cell size at deeper depths as in shallower waters. When the data have passed initial quality control, they are forwarded to a first “crude gridding” where all new data are incorporated together with the existing data (Fig. 3).

### Gridding, quality control and additional processing

The IBCAO grid is released in multiple versions, of which the official releases are “major versions”. Between these, several internal versions are generated as datasets are added, updated or removed. Additionally, the concept of grid “variants” is implemented in the grid compilation procedure, producing multiple grid variants of increasing quality for each internal version. These are, firstly, the “crude” grid, which includes all datasets regardless of quality; secondly, the “draft” grid, which includes partially processed data; and thirdly, the “final” grid. The first crude gridding is made for all existing data, including new data that have only undergone the initial individual review and processing. This gridding aims to determine if additional post-processing is required, using the tools available in Qimera or Caris. Some issues are first revealed when the datasets are merged and gridded together and compared to each other, with vertical offsets being an example. Other issues that may be revealed only when datasets overlap each other include sets of outliers that initially were assumed to represent real seafloor features. Processing may thus involve the removal of outliers, sections interfering with other datasets, and corrections of systematic vertical offsets. If datasets of relatively poor quality are found to be in conflict with other observations, they may be partially or completely removed.

We also analyse how well the dataset interacts with previously included contour data or grid steering points. Contours are digitised bathymetric data, typically from early navigation, exploration, or scientific charts, and may be inconsistent with newly measured data. New datasets may permit the complete removal of older contour lines from the grid or, if depth data are sparse, adjusting the contours to fit the new data. Steering points are artificially inserted data to guide the gridding algorithm on how to best produce a gridded surface. A classical case when steering points may be needed is in narrow fjords with few or no depth data points available. The gridded spline surface may in these cases produce landfilled fjords if it is fitted between the coastlines on both sides of the fjords without any constraining depth data in between. A few strategically inserted depth points will guide the spline surface, ensuring that the fjord remains a water-filled area. If the steering points for a specific region are shown to be incompatible with new data, they are removed. The general goal is to remove as many digitised contours and inferred steering points as possible from the compilation.

The draft gridding following the crude gridding, includes the updated datasets after they have been post-processed to account for issues found by analysing the results of the crude gridding (Fig. 3). Note that it often takes several iterations of gridding before all issues are addressed. In addition, the dataset prioritisation value needs to be assigned, and potentially later adjusted, to control the priority order when datasets overlap as well as deciding whether the dataset has a resolution and quality enough to be merged on top of the low-resolution base grid in the remove and restore process described further below. Furthermore, we may decide that some datasets need to be upsampled if they have irregular data gaps. Moreover, the processing status and a short description describing the remaining issues are added to the metadata.

When a dataset is found to be satisfactory, it is approved and entered into the final IBCAO gridding where the aim is to include only datasets with no major issues remaining. It should be noted that when a new dataset is entered into the draft grid, an additional control is performed on all previous datasets which come into contact with the new data and an evaluation of the gridding prioritisation order is performed. As previously mentioned, the new datasets may reveal undiscovered issues with older datasets and they may also be of higher quality than data previously located in the same area. In these cases, the new data are permitted to supersede previously included data, controlled by the prioritisation value.

### Gridding procedures

**Fig. 4:**
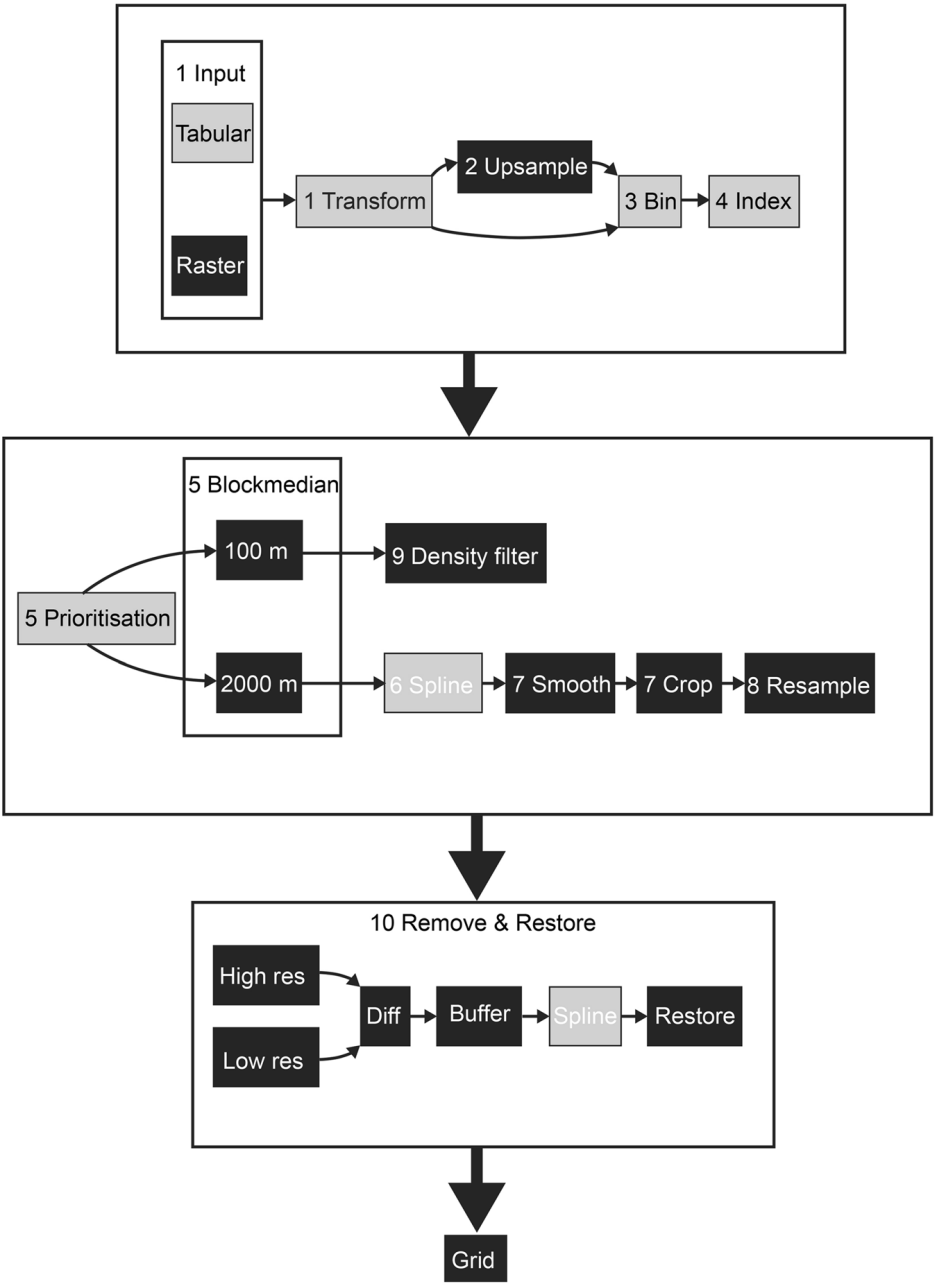
The gridding procedure shown schematically with all included main steps described in the text. Lightly shaded boxes represent tabular calculations using Pandas, dark shaded boxes represent raster calculations using NumPy whereas intermediate-shaded boxes represent interpolation using PyGMT. Note that this procedure is implemented for the “crude”, “draft” and “final” gridding as shown in Fig. 3.

**Fig. 5:**
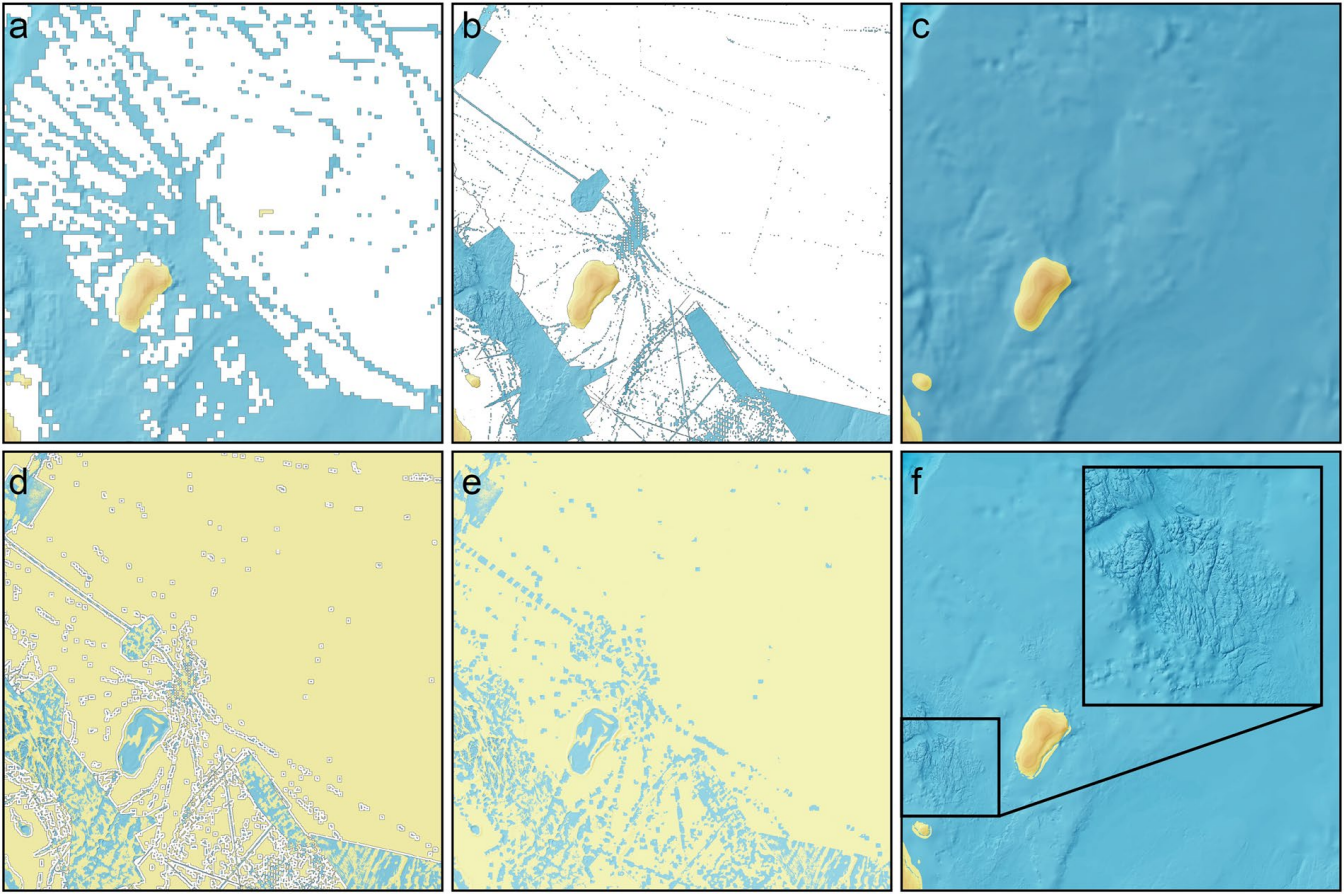
Visualisations of the outcome of some of the main gridding steps. The island is Kvitøya in eastern Svalbard. (**a**) Block median at 2,000 × 2,000 m of all data including both low- and high-resolution data after calculation step 5. (**b**) Blockmedian at 100 × 100 m after upsampling, showing the result of calculation step 5. (**c**) Low resolution 2,000 × 2,000 m interpolated, smoothed and resampled base grid produced using the blockmedian grid and the Generic Mapping Tools (GMT) spline in tension function^27^ in step 8. (**d**) Difference between (**b,c**), after applying a 20 grid cell empty buffer zone around high-resolution data in calculation step 10. (**e**) Interpolated difference values filling the buffer zones to make a smooth transition between high- and low-resolution data, also in step 10. (**f**) Restored final grid where the grid in **e** is added to **b**.

A schematic representation of the gridding procedure is shown in Fig. 4, and the outcomes of the key steps are depicted in Fig. 5. The gridding procedure implements the same routines to compile the “crude”, “draft” and “final” grid variants. The differences between the variants are only due to the selection of the included datasets, as previously described. The calculations are executed in a distributed computer environment. We utilise Amazon Web Services (AWS) and have automated the routine so that an incremental calculation can be triggered by a data contribution using the web form described above using AWS services, mainly Lambda, EC2, S3, DynamoDB and several additional services (IAM, EFS, ECR, CloudWatch, EventBridge Scheduler, etc.). We have also used computer resources available through the High-Performance Computing (HPC) system at the National Academic Infrastructure for Supercomputing in Sweden. The core of the gridding system is built in Python, primarily using the following libraries:

- NumPy for fast and optimised multi-dimensional array analysis,
- Pandas for managing tabular data structures,
- Dask used for distributed computing when data is larger than computer memory,
- SciPy including mathematical algorithms and
- PyGMT which is a Python interface to Generic Mapping Tools (GMT)^25^.

The gridding procedures are summarised here into ten calculation steps. The step numbers correspond to those shown in Fig. 4. The first five steps are performed using Pandas and the last five steps are computed mainly using NumPy.

### Step 1, reading and transforming

The first step consists of reading the depth data and its coordinates, SID and priority value into a point cloud table, currently consisting of more than 30 billion rows, each row representing one data point. Subsequently, all data points are transformed into a common coordinate system.

### Step 2, upsampling

The second step is performed for a subset of the data table, only containing rows with data points from high-quality datasets that are considered to be included in the high-resolution grid. Some of these datasets are individually upsampled to 100 × 100 m to eliminate data gaps, using the median algorithm described below, converting to raster mode and upsampling using a bilinear interpolation algorithm.

The third, fourth and fifth steps described below are performed on both data tables, i.e. the main table including all data as well as the high-resolution/high-quality subset data table.

### Step 3, binning

In the third step, the data points are binned into 100 × 100 m grid cells. Additionally, an index value is assigned to each data row representing the grid cell in which the data point is located, calculated from the XY coordinates using a simple integer division.

### Step 4, indexing

Step four consists of indexing the data tables, which involves sorting them by the assigned index value of each row. The purpose of this is to speed up the subsequent steps and to co-locate data points in the same grid cell. This is a computationally expensive and non-trivial operation for large data tables, particularly when the data are distributed among multiple machines.

### Step 5, blockmedian

In step five the assigned priority values for the datasets are used to find the data points with the highest priority value in each grid cell and to reject points with lower priority. The median depth value is subsequently found for the data points that have the highest priority. In the specific case when there is an even number of points (*n*) having the highest priority, the median would strictly be the average between the two depth (*Z*) values in the middle of the sorted data list according to the standard median definition

2 $$median(Z)=\frac{{Z}_{(n/2)}+{Z}_{((n/2)+1)}}{2},\text{where}\,n\,\text{is even}.$$

However, since we wish to stay as close to the original observed depth values as possible, we select the shallowest of the two centre values to represent the median of the grid cell instead of calculating the average. The reason for selecting the shallowest rather than the deepest can be considered somewhat arbitrary, although it should be noted that standard practice for producing any bathymetric product to be used for navigation, involves selecting the shallowest value. However, we stress that IBCAO is not a product to be used for navigation. There are cases where there are several data points with the highest priority having the exact same depth values. In this case, the data point with the highest SID (Source ID; a unique number given to each dataset) is selected to ensure that we always utilise the most recent dataset, even if the priority is the same. Note that even if there are more depth points with lower priority available than points with higher priority, the latter will be used. Now, only one value is selected for each non-empty grid cell and the data table can be transformed into a simple two-dimensional cartesian equirectangular raster consisting of grid cells. Two grids are created, one for depth values and a second for SID values. This is performed twice, once for all data including both high- and low-resolution data at a grid-cell size of 2,000 × 2,000 m (Fig. 5b) and at a grid-cell size of 100 × 100 m for the high-resolution data, upsampled if required (Fig. 5a). From this step and onwards only raster data are considered utilising NumPy mentioned above. (Fig. 4.)

Two processing paths are now continued, one high-resolution (100 × 100 m) and the other lower resolution (2,000 × 2,000 m). The low-resolution path consisting of steps six to eight provides a base grid over which the high-resolution data are merged once gridded using the remove-restore procedure^26^, described below. The high-resolution gridded data are generated using the high-resolution path in step nine.

### Step 6, interpolating

Calculation step six consists of interpolating the low-resolution 2,000 × 2,000 m blockmedian grid in order to fill all empty grid cells using a spline in tension function from GMT^27^, setting its tension factor to 0.34, after extending the domain size slightly to avoid edge artefacts.

### Step 7, smoothing

In step seven the interpolated grid is smoothed and cropped to the final domain. The smoothing is computed using a two-dimensional discrete convolution algorithm provided by the SciPy library. The weights kernel used by the convolution is 3 × 3 grid cells large (implying 6,000 × 6,000 m) and defined by a normalised cosine function.

### Step 8, resampling

In step eight the smoothed low-resolution grid is resampled to 100 × 100 m using bicubic interpolation (Fig. 5c). The resulting grid will be used later in step ten for the Remove and Restore procedure as the low-resolution component.

### Step 9, density filtering

Calculation step nine consists of determining whether an area is covered enough by data points to be included in the high-resolution component in the Remove and Restore procedure described in step ten. This is done by applying a density filter that rejects sparse data points if less than 30% of the grid cells in a 1,000 × 1,000 m area contain data values.

### Step 10, Remove and restore

Step ten is the last calculation step consisting of the Remove and Restore procedure (Fig. 5d). First, a cell-by-cell difference between the low and high-resolution grids is calculated, then an empty buffer zone is applied around the original high-resolution data (Fig. 5e). This buffer zone makes a smoother transition between the high-resolution data and the lower-resolution base grid as the spline function interpolates values in the buffer zone. The resulting grid is then interpolated using the GMT spline in tension function. The difference grid is thereafter merged in the Remove and Restore procedure on top of the low-resolution grid (Fig. 5f). This last step constitutes the so-called “restore” part of the algorithm.

We produce the final IBCAO 5.0 DTM at a resolution of 100 × 100 m, although it should be noted that this high resolution is only properly supported by depth measurements where the source data density has this spatial resolution or higher. In other areas, the interpolated low-resolution grid may provide a visually pleasing smooth bathymetry but will be composed of less accurate depths. In addition to the DTM, an SID is produced for each gridding. The SIDs for the grid cells provide a link to the metadata stored in the AWS Dynamodb database. The geographic 15 × 15 arc seconds GEBCO grids for the Arctic Seabed 2030 region are produced along with the Polar Stereographic grid using the same routines, although with the data points projected to geographic latitude and longitude coordinates.

### Incremental gridding

**Fig. 6:**
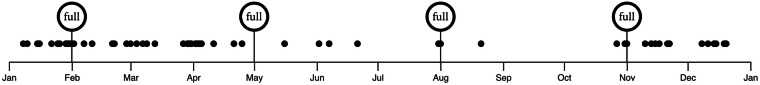
Illustration of how a substantially faster incremental gridding is frequently employed to ingest new data, while full gridding of the entire DTM regions is typically reserved for occasions when new DTM versions are published or significant edge effects are observed around included datasets.

While our focus lies in the Arctic region, our gridding procedure is designed to handle the entire World’s oceans of the size 400800 × 400800 grid cells and read > 30 billion depth points. We have divided the data tables and grids into data partitions since the total amount of data does not fit into the computer memory. The dividing scheme is varying throughout the entire gridding procedure. For the interpolation of the entire World ocean depth grid, it is divided into 160 × 160 overlapping partitions, each with the size of 2505 × 2505 grid cells representing 250,500 × 250,500 m. In order to perform faster calculations, we have implemented an incremental gridding mode that avoids re-calculating values that likely will not change when new data are added in a limited spatial area. Only the partitions where added data occur are re-gridded. Figure 6 shows a timeline illustrating how incremental gridding is performed rather frequently as new data are added. Full gridding is carried out quarterly or before the release of a major updated version of IBCAO/GEBCO. The full gridding will handle the edge effects that sometimes appear between the tiles.

### Source data

A list of all bathymetric source data, contributors, descriptions, and available references is provided together with the IBCAO 5.0 DTMs (10.17043/ibcao-5.0), which currently includes approximately 1400 datasets from approximately 78 sources and hundreds of contributing researchers and organisations. In addition, we have included here in the main article those citations of source data from this list that have Digital Object Identifiers (DOI)^28–179^. All source datasets used to compile IBCAO 5.0 are stored in XYZ or TIF-format in a repository, which contains a total of roughly 1550 datasets, although some datasets are composed of several merged surveys, some are part of the same survey and were divided to optimise the resolution versus depth (see Methods), and some have been deemed to not meet our quality requirements and are, therefore, no longer used. For this reason, the figure of 1550 datasets do not represent the exact number of datasets or sources used in IBCAO 5.0. Whenever possible, individual surveys are combined in the list of bathymetric source data (see 10.17043/ibcao-5.0) and are instead listed under the vessel used for the survey or their contributing organisation in order to shorten and simplify the table. Furthermore, in addition to the datasets within the IBCAO region, we also have access to datasets provided to Seabed2030/GEBCO that are primarily located in the north Pacific Ocean and the Atlantic Ocean, but extend into the Arctic IBCAO region. Approximately 500 datasets of this kind are included when producing IBCAO 5.0.

It should be emphasised that the IBCAO source data repository, hosted on AWS, is not open for public data download. For access to the source datasets, we refer to the original providers listed in our list of sources or the IHO Data Center for Digital Bathymetry (DCDB), where many of the included datasets are archived (https://www.ncei.noaa.gov/maps/iho_dcdb/). As previously mentioned, a unique SID is stored in the AWS metadata database, correlating each survey dataset with its metadata, as well as a TID, categorising the data according to the depth acquisition method listed in Table 2. Users may thus use the SID grid to find the datasets used in any given region. In addition, we store as much metadata related to each cruise or survey as can be determined, including the cruise or survey name, cruise report or survey publication, chief scientist, start and end date, start and end port, originator and provider, platform class, station name and ID, instruments used, and vertical and horizontal resolution.

Every dataset included in IBCAO 5.0 is typically handled in the manner described in the Methods section. Exceptions to these procedures are mostly made in cases where major gridded compilations with large spatial coverage are contributed to IBCAO. The largest gridded contributions included in IBCAO 5.0 are BedMachine Greenland^19^ Version 5^180^, providing bathymetry for coastal waters surrounding Greenland and under-ice topography of the island at a gridded resolution of 150 m, the MAREANO project mapping the Norwegian Continental Shelf and EEZ at a grid resolution of 50 m^181^, the EMODnet bathymetry DTM covering much of European southern Arctic waters at a grid resolution of 115 m^182^, and NONNA-100, which provides a compilation of bathymetry data from a large number of cruises in Canadian waters at a grid resolution of 100 m.

The BedMachine Greenland compilation^19^, available from the National Snow and Ice Data Center^180^, is derived from a number of sources, including NASA’s Operation IceBridge and additional ice-penetrating radar surveys to determine ice thickness, multibeam and single beam data on coastal-water bathymetry, as well as topography measurements to determine land elevation. In IBCAO 5.0, BedMachine has been updated to Version 5 from Version 3 which was used in the compilation of IBCAO 4.0. Whenever possible, the original bathymetry source data used in BedMachine has been acquired and entered into the IBCAO 5.0 to ensure that the original data and resolution is included in the compilation as well.

The algorithm employed to compile BedMachine is optimised to create a seamless transition at the ice/ocean interface and makes use of the subglacial topographic information from ice-penetrating radar^19^. In IBCAO 5.0, BedMachine is therefore used primarily along the Greenland coast. At a distance of about > 50 km from the coast, the IBCAO compilation algorithm is used instead. If new data are included close to the coast where BedMachine is used, they are blended into BedMachine by using the remove-restore procedure and an inferred buffer zone at a distance of 1 - 2 km surrounding the cruise track (See Methods for an explanation of remove and restore).

In IBCAO 5.0, the MAREANO bathymetric compilation of data from Norwegian waters was updated in April 2023, while IBCAO 4.0 included the September 2019 update.

EMODnet Bathymetry is a European Union project aimed at collecting bathymetric data from numerous European contributors to compile and publish a DTM of European waters every two years. The EMODnet DTM used in IBCAO 5.0 was updated from the 2018 version used in IBCAO 4.0 to the latest version available from 2022. It incorporates data from over 16,360 bathymetric surveys provided by 49 data providers across 24 countries. It should be noted that Stockholm University is a part of the EMODnet project consortium with responsibility for Arctic waters. Consequently, we aim to synchronise IBCAO and EMODnet compilations, ensuring that all new data included in IBCAO 5.0 are also integrated into any subsequent release of EMODnet.

The NONNA-100 (Non-Navigational) dataset, published by the Canadian Hydrographic Service, comprises bathymetric surveys collected using various mapping methods (see source data; 10.17043/ibcao-5.0). For IBCAO 5.0, the NONNA-100 compilation was updated to the 5 September 2023 version, replacing the 11 October 2018 version used in IBCAO 4.0. To provide better statistics for the coverage of the various TIDs, an algorithm was specifically developed to split the NONNA-100 dataset into smaller subsets containing data only from a specific TID. This segmentation was facilitated using unique identification numbers assigned to each datapoint in the NONNA-100 dataset, detailing the cruise and mapping method. Consequently, IBCAO 5.0 incorporates six datasets derived from the NONNA-100 data, each based on a distinct mapping method.

**Fig. 7:**
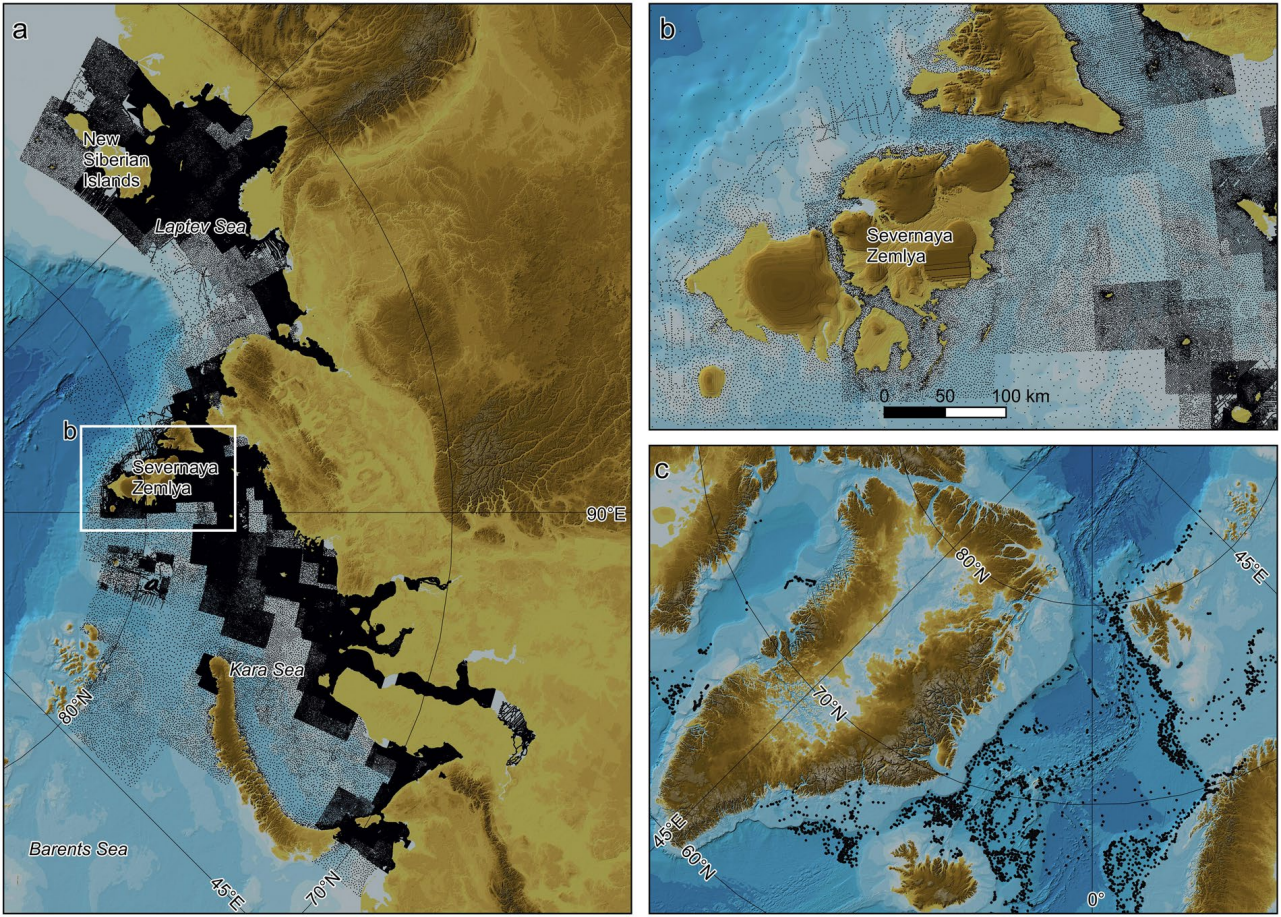
Examples of new major depth sources in IBCAO 5.0. (**a**) Soundings digitised from 150 published Russian navigational charts. (**b**) Close-up showing the sounding density around Russian Severnaya Zemlya. (**c**) Depths from seafloor groundings of Argo Floats.

A major new source for IBCAO 5.0 is 534505 soundings digitised from 150 published Russian navigational charts provided by the company East View Geospatial™. These soundings cover the Kara and Laptev seas, and the area surrounding the New Siberian Islands (Fig. 7a,b). Another new source is depths from seafloor groundings of Argo floats (Fig. 7c). These depths are derived from the measured pressure at the recorded point of grounding^183^. Although the Argo groundings cover a rather well-mapped part of the IBCAO 5.0 DTM, they show the potential of using depths derived in this manner for other, more sparsely mapped regions of the World Ocean.

Since the publication of IBCAO 4.0, numerous cruises and surveys across the Arctic Ocean have been added to the compilation. Multibeam surveys from research vessels (RV), such as the RV Araon, RV Knorr, RV Mirai, RV Maria S. Merian, RV Polarstern^184^, RV Sikuliaq, USCG Healy and IB Oden, are now used in the hundreds, providing significantly improved coverage in typically sea-ice covered regions of the Arctic Ocean. Major additions have been made to the compilation of coastal waters and fjords around Greenland and Svalbard in particular. Minor additions from various smaller surveys have also been made across the Arctic, providing further data in previously unmapped regions.

## Data Records

The IBCAO 5.0 DTM including the TID and SID grids are available for download from the GEBCO website (https://www.gebco.net/) and the Bolin Centre for Climate Research data repository (10.17043/ibcao-5.0)^185^. The DTMs we provide in netCDF and GeoTiff formats are compatible with popular GIS platforms such as QGIS and ArcMap. The Polar Stereographic projection of the DTMs is identified by code 3996 of the European Petroleum Survey Group (EPSG: https://epsg.io/), searchable via “IBCAO” or the EPSG code. The Polar Stereographic projection’s true scale is set at 75°N, with coordinates referring to the horizontal datum of WGS 84. The ‘x’ and ‘y’ variables refer to the grid-cell positions, along the x and y axis, in Polar Stereographic projected coordinates in metres. The ‘z’ value refers to depths (negative values) and heights (positive values) in metres below and above Mean Sea Level (MSL), respectively. However, it is important to note that not all bathymetric source data are properly vertically referenced to MSL, with many lacking metadata information on the vertical datum. In the TID grid, ‘band 1’ values signify the TID code corresponding to the data type used to derive the DTM cell value (Table 2), while ‘band 1’ values in the SID grid provide a unique link to the source data. For users who want to get an idea of the quality of depth data within a particular area of IBCAO 5.0, we advise the utilisation of the TID grid in conjunction with the DTM. This combination permits the identification of the type of source data used to derive the depth of any given grid cell, which will give insights into the reliability and accuracy of the depth information.

## Technical Validation

### Differences between IBCAO 4.0 and 5.0

**Fig. 8:**
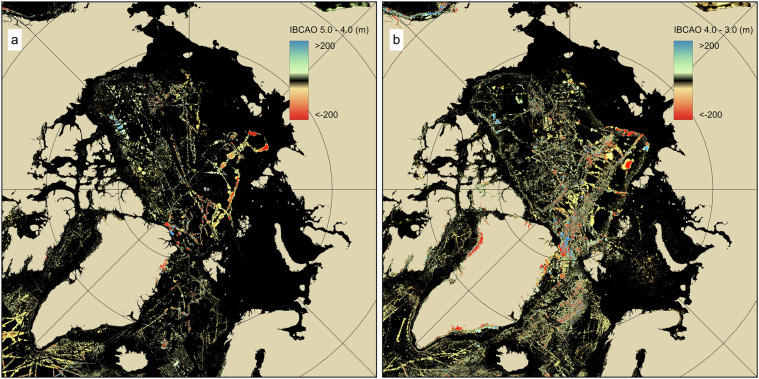
Comparison between different versions of IBCAO by subtracting one grid from the other. Black to dark colours show no or minor changes. (**a**) IBCAO 5.0 - 4.0. (**b**) IBCAO 4.0 - 3.0.

Significant progress has been made by the IBCAO project in mapping large sections of the Arctic Ocean and we no longer encounter entirely new shapes or discrepancies in major features such as the largest ridges. For example, the Lomonosov Ridge around 86°N 150°E displayed a completely different form in IBCAO 1.0 compared to its representation in the GEBCO Sheet 5.17^186^. Updates in the morphology of geological features from IBCAO 4.0 to 5.0 are less marked than those from 3.0 to 4.0 (Fig. 8), and we instead primarily see updated coverage in areas with known features but poor mapping. This is an expected trend, however, with only 25% of the Arctic now mapped, it also implies that our additional mapping is often in those places where some previous data existed.

**Fig. 9:**
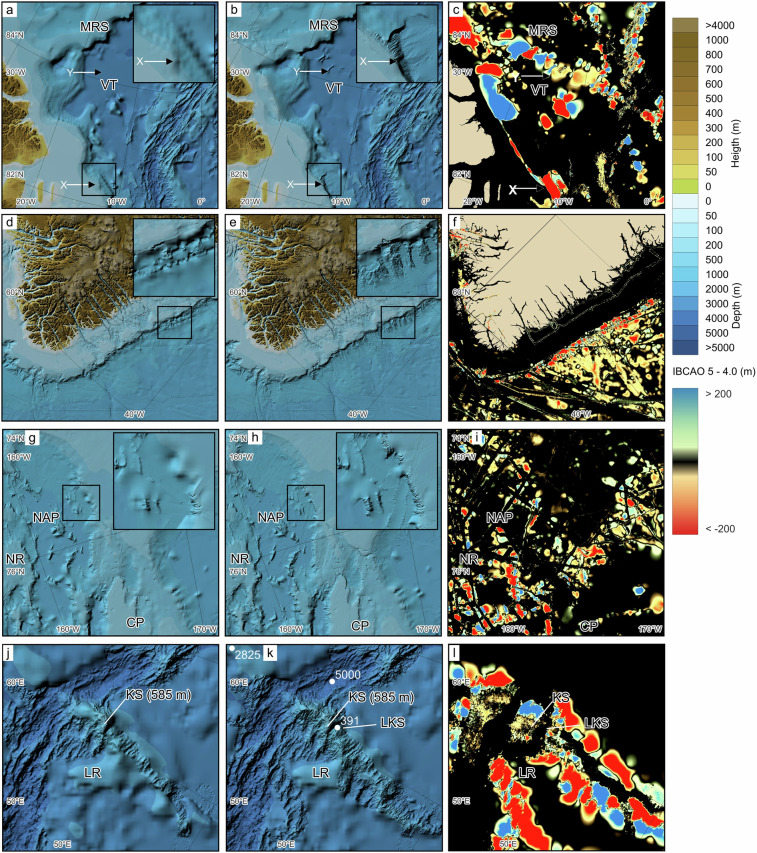
Comparison between IBCAO 4.0 and 5.0 in four selected areas. Locations of the areas are indicated in Fig. 1. The left column depicts IBCAO 4.0, the middle column shows IBCAO 5.0, and the right column displays the depth difference between the two (IBCAO 5.0 - 4.0). Colours toward blue indicate that IBCAO 5 is shallower than IBCAO 4.0, whereas colours towards red show the opposite. (**a**–**c**) Area north of the North Greenland continental margin and adjacent deep waters. X marks the significant change from IBCAO 4.0 to 5.0 in the portrayal and location of the continental shelf break and slope. Y shows where new multibeam bathymetry reveals a texture of the Voronov Terrace (VT). MRS = Morris Jesup Spur. (**d**–**f**) Southeast Greenland continental slope where new multibeam data reveal a typical slope morphology dominated by canyons. (**g**–**i**) Section of the Chukchi Borderland comprising the Northwind Ridge (NR), Northwind Abyssal Plain (NAP) and Chukchi Plateau (CP). Here several new multibeam tracks reveal steeper slopes surrounding the NAP. (**j**–**l**) The Langeth Ridge (LR) forming a part of the extensive Gakkel Ridge; the only active spreading ridge in the Arctic Ocean, which has the World ocean’s slowest spreading rates varying between about 6 and 13 mm/year^193^.

Even so, as comprehensive mapping is still lacking in several areas of the Arctic, there are some substantial changes in how IBCAO 5.0 depicts the seafloor morphology compared to 4.0. Four selected regions are highlighted in Fig. 9 for brief discussion. The first concerns one of the most sparsely mapped areas in the central Arctic Ocean. For example, the North Greenland continental margin and adjacent deepwater area, where the Morris Jesup Spur, also referred to as Morris Jesup Rise, extends roughly 240 km northward into the Amundsen Basin (Fig. 9a–c). Additional multibeam tracks added here since IBCAO 4.0 reveal significant inaccuracies in mapping the continental shelf and slope morphology. The newly added multibeam bathymetry for IBCAO 5.0 from the German Research RV *Polarstern* expedition PS115/1^187^ shows a continental slope with a more conventional appearance, characterised by smaller submarine canyons (see X in Fig. 9b). In this region, IBCAO 4.0 relied solely on gridded digitised contours from bathymetric maps, specifically the Russian paper chart “Bottom Relief of the Arctic Ocean” published in 2001^188^.

We have refrained from modifying the bathymetry beyond correcting inaccuracies revealed by new data, as discrepancies in one segment of a published contour map do not necessarily imply inaccuracies in others. The primary challenge with historic maps commonly lies in the lack of information regarding the underlying source data. However, until new depth data become available, historic maps remain the sole resource for certain areas of the Arctic Ocean. New multibeam data from the Swedish icebreaker (IB) Oden, acquired during the Synoptic Arctic Survey (SAS) Expedition in 2021^189^, suggests that the apparently smooth Voronov Terrace in IBCAO 4 likely has relief inherited from the seafloor spreading that opened the entire Amundsen Basin (see Y in Fig. 9a,b).

Moving southward along Greenland’s eastern continental slope to approximately 61°N, recent bathymetric data reveal the influence of slope processes, leading to the formation of prominent canyons and sedimentary fan-drift bodies in this region. These features were not identified in IBCAO 4.0 (Fig. 9d,e) and may suggest that similar morphological elements characterise much of the slopes offshore of Greenland’s continental shelf, although observations are limited to areas where multibeam bathymetry data are available.

Shifting focus to the Amerasian Basin side of the central Arctic Ocean, where the Chukchi Borderland extends northward from the shallow continental shelf. The borderland includes the Chukchi Plateau and Northwind Ridge, separated by the Northwind Abyssal Plain (see Fig. 9g,h). This example demonstrates how successive multibeam tracks acquired during transits reveal the seafloor morphology. The slopes surrounding the Northwind Abyssal Plain are notably steeper and feature the formation of relatively small canyons, not previously seen in IBCAO 4.0.

A fourth highlighted example is the Langseth Ridge located at approximately 86°30’N 61°30’E. The ridge features several peaks, including one hosting the shallowest point of the Gakkel Ridge known as Karasik Seamount (Fig. 9j,k). This peak reaches a depth of about 585 m according to multibeam bathymetry acquired by RV *Polarstern* in 2016^190^. However, due to IBCAO’s nature as a gridded DTM based on block-median values of 100 × 100 m grid cells derived from the underlying source data, the depth is slightly deeper in the grid (587 m). Adjacent to Karasik Seamount, another shallower seamount reaching a depth of 391 m is marked on the Russian bathymetric map from 2001^188^. This peak was initially mapped during the Soviet Northern Fleet Hydrographic Expedition in 1965 from a drift ice station and proposed to be named the Leninskiy Komsomol Seamount after the Russian submarine “Leninskiy Komsomol,” which first surfaced at the North Pole in 1964 (GEBCO, Undersea Feature Names Gazetteer: https://www.ngdc.noaa.gov/gazetteer/). The name was officially adopted by GEBCO’s Subcommittee for Undersea Feature Names (SCUFN) in 2002. The RV *Polarstern* expedition PS101 in 2016 had as one of its priorities to map the Langeth Ridge and resolve the configuration of the peaks there^190^. However, the existence of the Leninskiy Komsomol Seamount at the marked location could not be verified. This is not surprising, given the significant challenges associated with conducting mapping from ice-drift stations in the 1960s, a period predating the availability of accurate Global Navigation Satellite Systems (GNSS) and narrow beam echosounders.

### Gridding algorithm

**Fig. 10:**
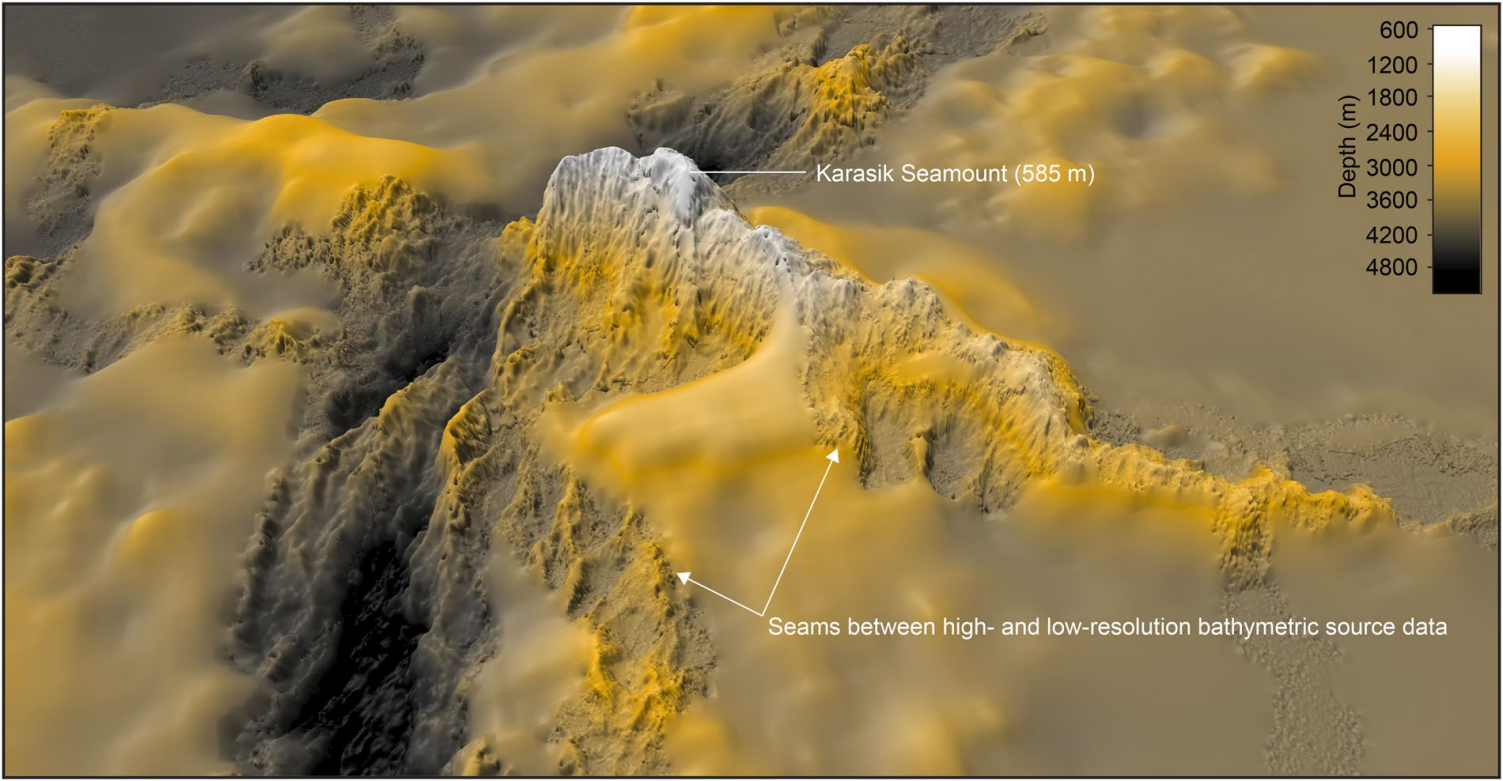
3D view of the Langseth Ridge area shown in Fig. 9j-l. The visualisation illustrates an example of when our gridding algorithm works exceptionally well in generating smooth seams between high- and low-resolution source data.

When our gridding algorithm works optimally, high-resolution bathymetry, such as multibeam data, seamlessly integrates with coarser gridded surfaces generated by applying the spline-in-tension interpolation method to sparse soundings and/or digitised contours. The Langseth Ridge is a prime example, where high-resolution bathymetry reveals details of the ridge morphology and the transitions between resolutions appear visually seamless (Fig. 10). However, in certain areas, the integration of high and low-resolution bathymetry is less smooth, resulting in more pronounced and visually unappealing seams, a topic we delve into further when addressing specific artefacts below.

### Errors

All bathymetric source data incorporated into the compilation of the IBCAO 5.0 DTM carry their associated errors into the gridded product. We utilise the TID grid as a primary indicator of the reliability of IBCAO 5.0 in any given region, as it offers the best indication of uncertainty available. For instance, where TID codes in Table 2 indicate the use of direct depth measurements, such as TID = 11 for multibeam, the grid offers a more precise representation of the seafloor compared to instances where indirect methods were employed, such as TID = 42 for depth values derived from a bathymetric contour dataset. A more detailed assessment of uncertainty necessitates going into source identification, where the sources utilised for each grid cell can be traced back to a specific survey.

Quantifying the uncertainty of a bathymetric DTM derived from a combination of sources is not straightforward. Various statistical methods, such as Monte Carlo simulation, have been employed to analyse how the random error inherent in the source data propagates into the final grid^191,192^. This random error affects the standard deviation of grid-cell depths, particularly in regions where bathymetry changes rapidly, such as along steep slopes, where errors in navigation will have very large effects. However, propagating this random error into the final grid requires assigning a random error to each individual source dataset, a monumental task that we have not been able to undertake at this stage for all > 1500 datasets used.

**Fig. 11:**
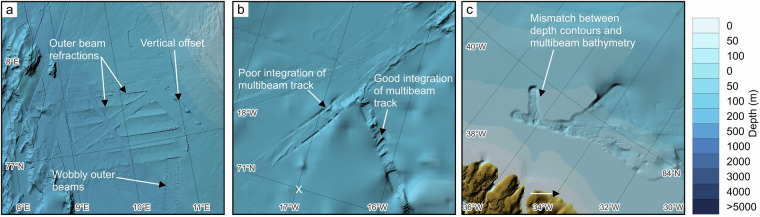
Examples of issues encountered in IBCAO 5.0. (**a**) Area west of Svalbard with examples of refractions and wobbly outer beams most likely due to poor sound velocity control during data acquisition. (**b**) An example east of southern Greenland where the gridding algorithm failed to optimally merge the multibeam bathymetry with the surrounding grid based on sparse source data. (**c**) An example of digitised depth contours poorly matching multibeam bathymetry.

Whereas the general effects of random errors may not be easily discernible in a bathymetric DTM, other errors inherited from the data are readily apparent. These include artefacts from poor control of sound velocity in acquired multibeam bathymetry, resulting in refraction of outer beams, or vertical offsets due to the use of different vertical reference levels^24^. Unfortunately, information about the vertical reference level is commonly not described in the metadata. Furthermore, our gridding algorithm is not always as successful as the example shown in Fig. 10. There are several areas where the edges between different datasets are visually unappealing. Figure 11 shows some typical examples of these issues. We are working with the source data on correcting these types of errors where possible.

## Usage Notes

The primary uses of the IBCAO DTM have remained consistent since the initial release of Version 1.0. Therefore, the “Usage notes” for Version 5.0 closely resemble those of IBCAO 4.0^14^. The most widespread applications include map-making and geospatial analyses using GIS software. The IBCAO products are not intended for use for navigation or any other purpose involving safety at sea. While as much care as possible has been taken to ensure the highest achievable quality of the IBCAO 5.0 DTM, there are always limits to how accurate and reliable the underlying data are. Visual analysis and quality control are performed on every dataset entered into the grid, though there are cases where the quality of the data is difficult to assess, particularly in regions with poor coverage. Furthermore, IBCAO 5.0 makes use of bathymetric data from a vast number of sources of different qualities, resolutions, and coverages, as well as interpolation of measured data between non-mapped grid cells and upsampling of data with a lower resolution than that of the published grid. Therefore, the accuracy of the IBCAO 5.0 DTM varies significantly depending on the underlying source data and its resolution, and we cannot guarantee that the Arctic seafloor is as accurately mapped as it may appear in the grid. In summary, the accuracy of the IBCAO 5.0 DTM cannot be guaranteed and the authors and contributors involved in its production and publication cannot accept responsibility for any resulting loss, injury, or damage arising from the use of the DTM.

The Polar Stereographic coordinates can be converted to geographical positions using the GMT command mapproject with the following parameters:

*mapproject [input_lonlat] -R-180/180/0/90 -Js0/90/75/1:1 -C -F > [output_ xy]*where *input_lonlat* is a table with longitude and latitude geographic coordinates and *output_xy* is a table with the resulting converted xy Polar Stereographic coordinates. The inverse conversion from xy to geographical coordinates is achieved by adding *-I* to the command above.

The GDAL command *gdaltransform* can also be used to convert between the Polar Stereographic and geographic coordinates by calling for the EPSG codes 3996 and 4326 (WGS 84 geographic):

*gdaltransform -s_srs EPSG:4326 -t_srs EPSG:3996*

The inverse conversion is simply achieved by swapping the order of the EPSG codes.

## Data Availability

The procedures involved in compiling the various bathymetric source data into the IBCAO 5.0 DTM, as described in the Methods section, are based on open-source routines. These include the spline in tension gridding algorithm provided by GMT (https://www.generic-mapping-tools.org/) and various Python tools. Codes of the calculations are available from the Bolin Centre git (https://git.bolin.su.se/rez/seabed2030-calc).
